# Metal oxide semiconducting nanomaterials for air quality gas sensors: operating principles, performance, and synthesis techniques

**DOI:** 10.1007/s00604-022-05254-0

**Published:** 2022-04-21

**Authors:** N. A. Isaac, I. Pikaar, G. Biskos

**Affiliations:** 1 Fachgebiet Nanotechnologie, Technische Universität Ilmenau, 98693 Ilmenau, Germany; 2 School of Civil Engineering, The University of Queensland, Brisbane, QLD 4072 Australia; 3 Climate and Atmosphere Research Center, The Cyprus Institute, 2121 Nicosia, Cyprus; 4 Faculty of Civil Engineering and Geosciences, Delft University of Technology, Delft, 2628 CN The Netherlands

**Keywords:** Air quality monitoring, Metal oxides, Chemiresistive gas sensors, Sensitivity, Selectivity

## Abstract

To meet requirements in air quality monitoring, sensors are required that can measure the concentration of gaseous pollutants at concentrations down to the ppb and ppt levels, while at the same time they exhibiting high sensitivity, selectivity, and short response/recovery times. Among the different sensor types, those employing metal oxide semiconductors (MOSs) offer great promises as they can be manufactured in easy/inexpensive ways, and designed to measure the concentration of  a wide range of target gases. MOS sensors rely on the adsorption of target gas molecules on the surface of the sensing material and the consequent capturing of electrons from the conduction band that in turn affects their conductivity. Despite their simplicity and ease of manufacturing, MOS gas sensors are restricted by high limits of detection (LOD; which are typically in the ppm range) as well as poor sensitivity and selectivity. LOD and sensitivity can in principle be addressed by nanostructuring the MOSs, thereby increasing their porosity and surface-to-volume ratio, whereas selectivity can be tailored through their chemical composition. In this paper we provide a critical review of the available techniques for nanostructuring MOSs using chemiresistive materials, and discuss how these can be used to attribute desired properties to the end gas sensors. We start by describing the operating principles of chemiresistive sensors, and key material properties that define their performance. The main part of the paper focuses on the available methods for synthesizing nanostructured MOSs for use in gas sensors. We close by addressing the current needs and provide perspectives for improving sensor performance in ways that can fulfill requirements for air quality monitoring.

**Graphical abstract:**

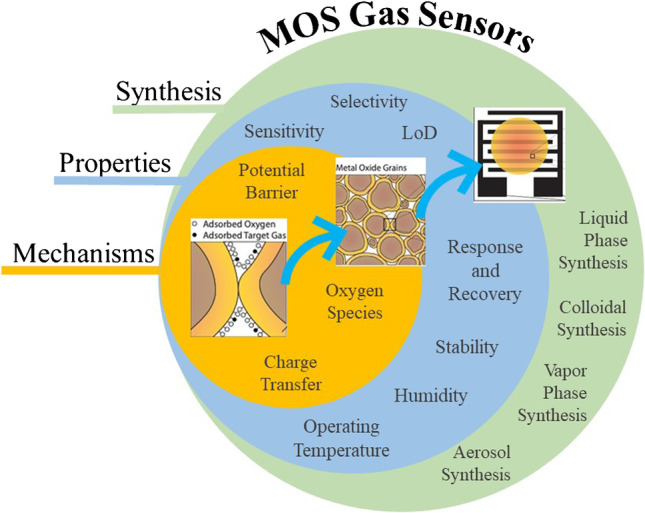

## Introduction

Air pollution is associated with negative effects upon human health [1–3], leading to some 7 million premature deaths per year worldwide as recent estimates from the World Health Organization (WHO) show [4]. Aside from its health impacts, air pollution also has detrimental environmental effects as among others it accelerates climate change, leads to acid rain, and changes soil chemistry that in turn affects plant growth and groundwater quality [5–9]. According to the Organization for Economic Co-operation and Development (OECD), the environmental impacts of air pollution lead to economic repercussions that are estimated in the order of multiple trillion US dollars per year globally [10].

Traditional air quality monitoring is carried out by analytical instruments installed at fixed stations. The location of these stations is selected based on the proximity to major air pollution sources, whether the resulting measurements would be representative for a wider region, and other practical factors such as availability of power and site safety. The number of air quality monitoring stations used in national networks varies depending on the size and the economic wealth of each country. For instance in the UK, which is a country that makes substantial investments in environmental monitoring, the Automatic Urban and Rural Network (AURN) has ~ 300 monitoring stations (1 station in ca. every 830 km^2^) to monitor common gaseous pollutants and particulate matter [11]. Even in London, where the need to map air quality is stronger due to the high population density, monitoring is carried out at 14 stations that cover ca. 1600 km^2^, yielding a coverage of 1 station in ca. every 110 km^2^ [12].

To determine the impacts of air pollution on human health and on the environment, we need to monitor the concentrations of the most harmful pollutants with a spatial coverage that is far higher compared to that of existing air quality monitoring stations. This can be achieved by using stationary and/or mobile platforms with integrated gas sensors that have a suitable limit of detection, small response times, and low cross sensitivity. Moreover, it is desired that sensors be made low-cost, energy efficient, and robust.

Gaseous pollutants can be either inorganic (e.g., CO, NO, NO_2_, SO_2_, and O_3_) or organic (volatile organic compounds; VOCs) molecules, and their concentration in the atmosphere can vary from a few ppt to a few tens of ppm. Depending on their operating principle, gas sensors can be categorized as: acoustic wave sensors [13], quartz microbalances [14], calorimetric sensors (also referred to as anemometers or pellistors) [15], electrochemical cells [16], and field-effect transistors [17]. Other categories of sensors include those that probe changes of the optical properties (transmission and/or reflection) [18] of the sensing material caused by absorption of the target gas molecules, or of the electrical properties (resistance, conductance, capacitance, and impedance) of the gas sensing material as a result of the adsorption of gas molecules onto their surface.

A promising category of sensors, which is increasingly being considered for air quality monitoring, are those that employ a metal oxide semiconductor (MOS) [19]. The resistance of MOSs can be highly sensitive to the type and number of adsorbed molecules on their surface. Compared to other types of materials, they have several advantages, including that they can be easily manufactured and implemented, and that they can yield sensors having adequate response (from a few seconds to minutes) and sensitivity for a wide range of target gases [20].

Patented in the 1970s, Taguchi demonstrated that tin oxide chemiresistors can be used as effective gas sensors [21]. Following that, a number of studies have provided techniques for improving sensor sensitivity, selectivity, stability, and response time, as well as for reducing fabrication cost [22]. At present, MOS chemiresistors are mostly used for industrial applications where the concentrations of the target gas molecules are relatively high (ppm or % levels). For example, Renesas Corporation, in Japan, produces industrial MOS H_2_ sensors which have a specified operating range from < 10 to 1000 ppm, whereas Figaro Inc., also in Japan, fabricates MOS gas sensors for measuring the concentration of H_2_S, NH_3_, and CO, among other gases, in the range of 10–1000 ppm.

Gas sensors that employ MOSs hold great potentials for applications in air quality monitoring due to their low cost and high portability, and thus significant efforts have been made towards optimizing them for sensing key gaseous pollutants. Although the majority of MOSs can exhibit changes in their resistance as a result of the adsorption of gaseous species on their surface, specific materials have been shown to be more appropriate than others for different target gases. For example, ZnO, SnO_2_, and TiO_2_ are commonly used for measuring CO [23], whereas WO_3_ is traditionally employed for NO_2_ sensors [24]. A number of techniques such as doping, nanostructuring, and mixing with other families of materials to produce composites have been employed to improve the specifications of the end sensors, including their sensitivity, selectivity, and response/recovery times.

Despite the immense  research efforts, use of MOS sensors for environmental monitoring have challenges: (a) their output signal should ideally not be affected by gases other than the target gas (i.e., they should have a high selectivity), (b) their sensitivity should be temperature and humidity independent, and (c) they should exhibit high repeatability. Recent advancements in nanomaterial synthesis have addressed a number of limitations of MOS gas sensors for application in air quality monitoring. This has led to a number of proof-of-concept studies for the development and implementation of MOS gas sensors with limits of detection down to 0.05 ppm for explosive markers [25], as well as for environmental monitoring of several key urban air pollutants, including nitrogen oxides (NO and NO_2_) and ozone (O_3_) [26], volatile organic compounds (VOCs) [27], sulfur oxides (SO_x_) [28], and carbon monoxide (CO) [29].

A number of review papers focusing on different aspects of MOS gas sensors have been published recently. Dey et al. [30] focused on the selectivity, sensitivity and stability of MOSs, whereas Ji et al. [31] provided an overview of the involved gas sensing mechanisms and Al-Hashem et al. [32] focused on the role of oxygen vacancies on the sensing mechanism of MOS materials and the overall sensor performance. Application of MOS gas sensor for air quality monitoring has also been reviewed [33]. Despite those efforts, however, an overview of the field addressing the operating principles, the performance characteristics and the methods for synthesizing MOSs for gas sensing applications is missing.

In this paper, following an extensive discussion on the operating principles and properties of gas sensors, we present a comprehensive literature review on the relevant state-of-the-art synthesis methods of MOS nanomaterials that can provide properties required for use in environmental monitoring. The last section gives future research and development directions for enabling industrial manufacturing of MOS gas sensors that can be employed in air quality monitoring.

## Operating principle and performance of MOS gas sensors

### Operating principle

**Fig. 1:**
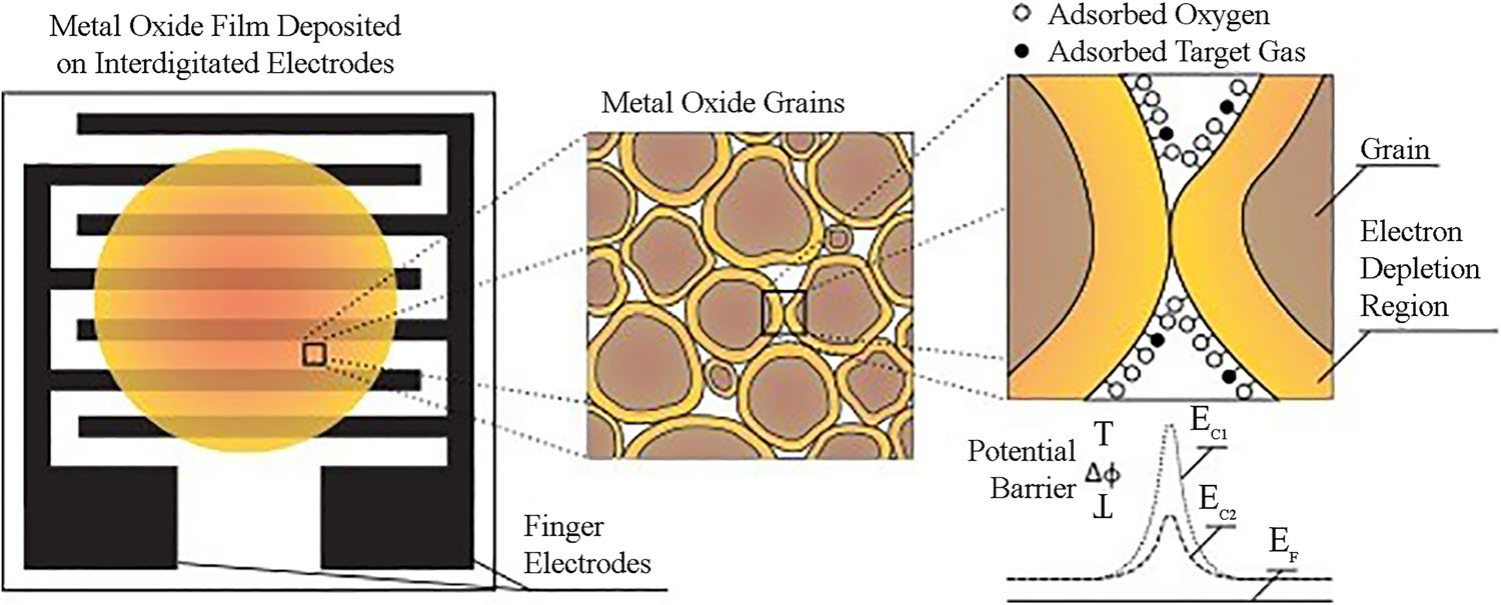
Schematic diagram showing a particulate-based thin film deposited on an interdigitated substrate, and the interaction of the grain boundaries after the gas species adsorb on the metal oxide surface. Left: Illustration of a chemiresistive gas sensor consisting of the metal oxide semiconducting nanomaterial (orange film) deposited on an interdigitated substrate with two finger electrodes providing connections to a circuit for resistance measurement; Middle: Microstructural characteristics of the MOS film showing the grain boundaries (orange layers), Right: Ambient oxygen species adsorb on the metal oxide surface, depleting the electrons from the conduction band throughout the material. This creates a space charge layer (depletion region), and consequently a barrier to the charge carrier flow at the grain boundaries. For an n-type particulate MOS gas sensor in the presence of adsorbed target gas molecules (e.g., NO_2_), the space-charge region widens and the conduction band bending of the material increases from E_C2_ to E_C1_. This bending creates an increase of the potential barrier to the path of the charge carrier (e.g., electrons), which transduces to a change in resistance of the MOS film measured by an external circuit connected to the interdigitated electrodes

Low-cost gas sensors typically rely on changes of the macroscopic properties of their sensing material upon exposure to different concentrations of target gases. To achieve good sensitivity, the materials are typically comprised of grains (i.e., particulate building blocks) that gently attach one another. Gas sensors employing MOS grains/particulates in the form of thin films rely on changes in the conductivity of the material caused by the adsorption of a number of target gas molecules on their surface, which is proportional to the concentration of the gas in the overlaying gas (cf. Fig. 1). Overall, the sensing mechanism of MOS gas sensors can be described in two steps: 1. the gas–solid interfacial interaction (referred to as receptor function), followed by 2. the transduction of this interaction to an electrical signal caused by the resistance change of the sensing material (called transducer function).

Two widely accepted mechanism for the receptor function have been proposed in the literature: namely the ionosorption and the oxygen vacancies mechanism [34]. In ionosorption, the chemisorption of oxygen species on the surface of the MOS is considered the primary sensing mechanism. In the oxygen vacancies mechanism, on the other hand, changes in the electrical properties of the MOS are induced by oxidation/reduction reactions that take place on its surface. In either case, the sensitivity and other properties of the sensors depend on factors influencing these surface reactions, including chemical interactions between the gaseous species in the sample and the sensing material, surface-modification and microstructures of sensing material, as well as environmental factors such as temperature and humidity.

Oxygen species (O_2_^−^, O^−^, O^2−^, O_2_) play a major role in the operating mechanism of MOS gas sensors. While molecular oxygen is stable below 150 °C, atomic species dominate at higher temperatures as oxygen ionizes to form molecular (O_2_^−^) and atomic (O^−^, O^2−^) species [35]. Adsorbed oxygen primarily modifies the charge distribution on the surface of the MOS. Formation of oxygen surface ions takes place as the adsorbed molecules extract electrons from the conduction band at the grain boundaries, creating an electron-depleted region that forms a space-charge layer that is manifested as a conduction band bending. The electron-depleted regions throughout the material create locally (at the grain boundaries) a potential barrier that affects the flow of charge carriers in the material [36].

The potential barrier of the electron-depleted regions is further increased when apart from the oxygen also the target gas molecules adsorb at the particulate MOS surface, as illustrated in Fig. 1. Depending on the type of the sensing material (i.e., whether it is an *n*- or a *p*-type semiconductor) and the target gas (i.e., whether it is oxidizing or reducing), the absolute change in material electrical resistance can vary substantially over several orders of magnitude. While an oxidizing gas would deplete the surface from the electrons, the reverse is true for a reducing gas. Depletion of conduction-band electrons causes a decrease of charge carriers in an *n*-type semiconductor, thereby increasing the resistance of the sensing material [37]. The opposite happens for a *p*-type semiconductor. In contrast to *p*-type, *n*-type nanomaterials do not exhibit any tendency to exchange their lattice oxygen atoms with the gas, making them more stable and thus more favorable as gas sensing materials [38].

The charge transfer mechanism between an *n*-type semiconductor and an electron depleting gas (e.g., NO_2_) can be described by the following reactions [39]:

**Table:** 

${\mathrm{O}}_{2}\left(\mathrm{gas}\right)\to {\mathrm{O}}_{2}^{-}(\mathrm{ads})$(for *T*_op_ < 150 ºC)	Reaction 1
${\mathrm{O}}_{2}+2{\mathrm{e}}^{-} \to 2{\mathrm{O}}^{-} (\mathrm{ads})$(for *T*_op_ > 200 ºC)	Reaction 2
${\mathrm{NO}}_{2} \left(\mathrm{gas}\right)\leftrightarrow {\mathrm{NO}}_{2}(\mathrm{ads})$	Reaction 3
${\mathrm{NO}}_{2}\left(\mathrm{ads}\right)+ {\mathrm{e}}^{-} \leftrightarrow {\mathrm{NO}}_{2}^{-}(\mathrm{ads})$	Reaction 4
${\mathrm{NO}}_{2}\left(\mathrm{ads}\right)+ {\mathrm{e}}^{-}\leftrightarrow \mathrm{NO}\left(\mathrm{gas}\right)+{\mathrm{O}}^{-}(\mathrm{ads})$	Reaction 5
${\mathrm{NO}}_{2}\left(\mathrm{ads}\right)+{\mathrm{O}}_{2}^{-}\left(\mathrm{ads}\right)+2{\mathrm{e}}^{-} \leftrightarrow {\mathrm{NO}}_{2}^{-}\left(\mathrm{ads}\right)+2{\mathrm{O}}^{-}(\mathrm{ads})$	Reaction 6
NO_2_ + M^x+^ $\to {\mathrm{M}}^{\left(\mathrm{x}+1\right)+}-$ ${\mathrm{NO}}_{2}^{-}$ $\to {\mathrm{M}}^{\left(\mathrm{x}+1\right)+}- {\mathrm{O}}^{-} +\mathrm{NO}$ (desorption)	Reaction 7
2 ${\mathrm{M}}^{\left(\mathrm{x}+1\right)+}- {\mathrm{O}}^{-} \to {\mathrm{M}}^{\mathrm{x}+}+ {\mathrm{O}}_{2}$ (desorption)	Reaction 8

The operating temperature (*T*_op_) refers to the surface temperature of the MOS, which defines the type of oxygen species present on its surface, as listed in Table 1, and consequently the response of the sensor. Reaction 1 ccurs when *T*_op_ is < 150 ºC, while Reaction 2 takes place at *T*_op_ > 200 ºC [40], inducing different surface chemical reactions with the target gas molecules.

Wang et al. demonstrated that *T*_op_ plays a major role in gas sensor sensitivity when this is < 180 ºC. This is because surface reaction kinetics are slow at this range of temperatures, becoming the limiting factor of the gas sensing mechanism. Target gas diffusion through the grain boundaries, which is another factor that affects the overall performance of the sensors, play an important role as *T*_op_ increases [41]. At higher operating temperatures, the surface reactions show faster kinetics and thus cease to be the rate determining factor. At *T*_op_ > 260 ºC, the diffusion rate of the target gas molecules becomes the limiting factor for gas sensing, indicating that the grain morphology, the nanostructure pore size, and surface to volume ratio can become important parameters defining the overall gas sensing mechanisms [41].

### Performance of MOS sensors

Sensor performance can be characterized by several parameters including the limit of detection, sensitivity, response and recovery times, selectivity, cross sensitivity, stability, and lifetime. These parameters are crucial for selecting the most appropriate solution depending on the application. Definitions of each of these parameters is provided in the following paragraphs.

#### Limit of detection (LoD)

Limit of detection is defined as the lowest concentration of the gas analyte that can be measured. The LoD is an essential parameter defining the suitability of any type of gas sensors, including those employing MOS sensing materials, especially when those are designed for air quality monitoring. Threshold limits of the main air pollutants set by the European Union (EU) and by the National Ambient Air Quality Standards (NAAQS) in the US range from a few ppb to a few ppm as shown in Table 2. Although results reported in the literature show that MOS gas sensors can operate below these limits (cf. cited references in Table 2), these systems are still at the research and development stage. Before assessing if and how these can systematically be employed in air quality monitoring, several parameters including their selectivity, robustness and cost-effectiveness need also be considered.

**Table 1:**
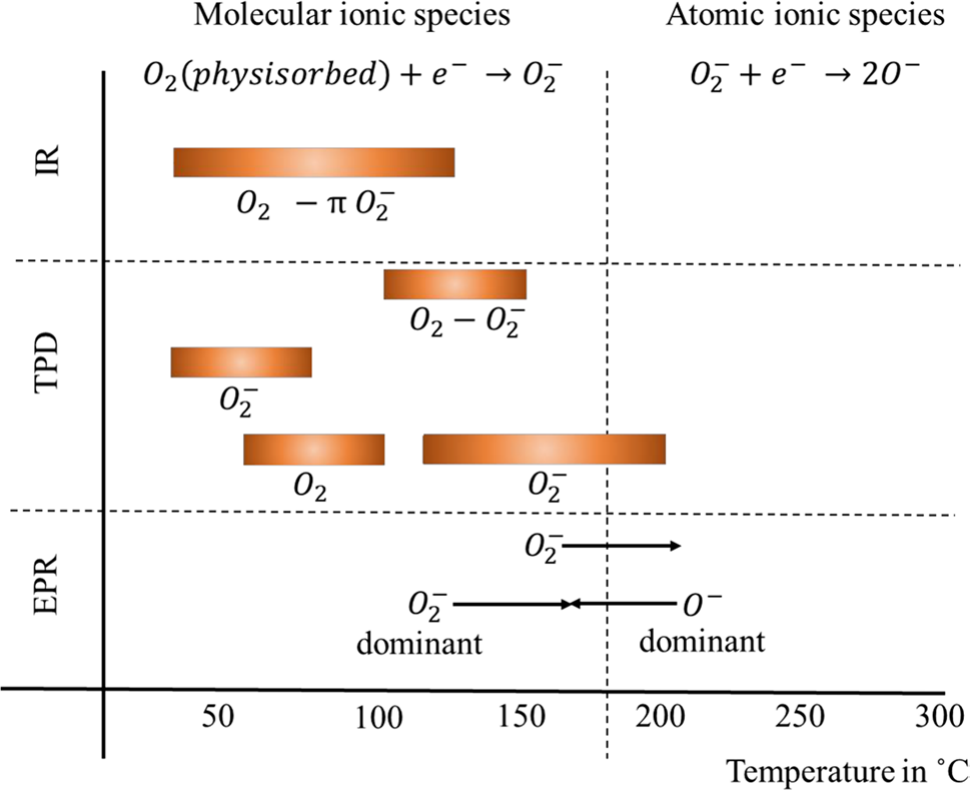
Oxygen species on SnO_2_ at various temperatures identified by Fourier-transform infrared (IR), temperature programmed desorption (TPD), and electron paramagnetic resonance (EPR). The dominant oxygen species changes from O_2_^−^ at lower temperatures towards O^−^ at temperatures around 175 ºC. Data extracted from Barsan and Weimar [42]

#### Sensitivity

**Fig. 2:**
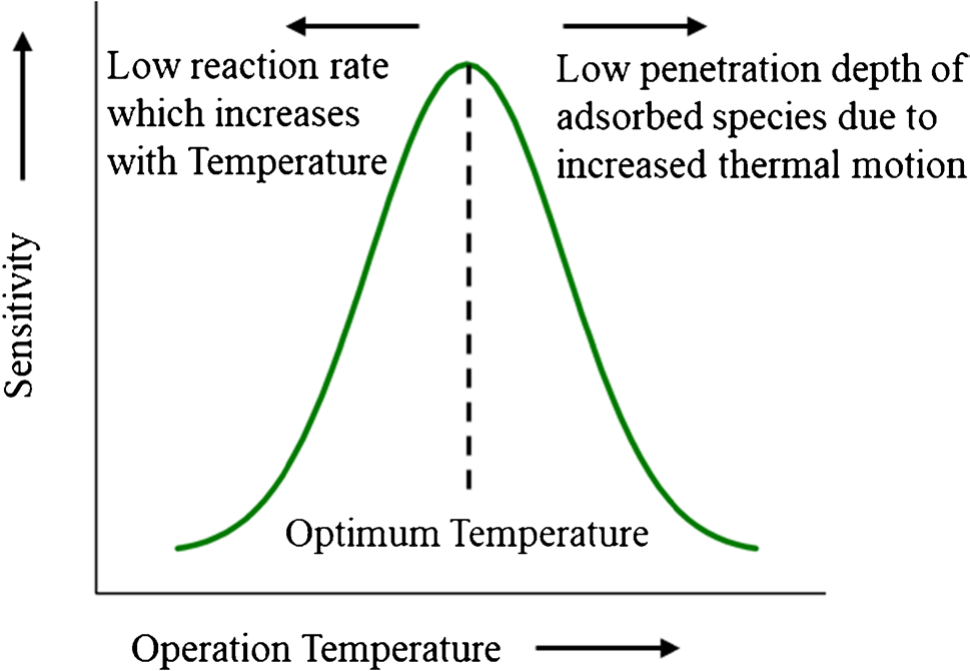
Dependence of the sensitivity of MOS gas sensors on the sensor operating temperature. An increase in temperature initially increases the adsorption (chemisorption and physisorption) of gas species. However, after a specific threshold (which varies depending on the MOS and the target gas molecule) the high thermal motion of the adsorbed species promotes desorption, which in turn decreases sensor sensitivity

As described in the “Operating principle” section, adsorbed species on the surface of thin-film MOSs affect the current that can pass through the nanostructure. Sensitivity represents the change in electrical signal (i.e., change in the resistance of the MOS) as a function of gas concentration. It is defined as the ratio of the response signal when the sensing material is exposed to the target gas analyte, to the response signal in the presence of air:

1 $$S = \frac{{R}_{g}}{{R}_{a}},$$

where *R*_*a*_ and *R*_g_ are respectively the resistance of the MOS in pure air, and air containing a fixed concentration of target gas. It should be noted here that sensitivity is a function of the temperature at which the MOS can be maintained (i.e., the operating temperature of the sensor), typically exhibiting a maximum/optimum value as illustrated in Fig. 2.

As the temperature increases, enhanced thermal motion of the target gas molecules leads to an increase of their diffusion to the bulk MOS. In addition, chemisorption is preferred over physisorption at lower temperatures, forming strong target gas-MOS surface chemical bonds that promote adsorption. This enhanced interaction increases the resistance changes of the MOS and thus the sensitivity of the sensor (cf. Fig. 2). As temperature is further increased, thermal motion of the adsorbed species increases desorption rate, which in turn decreases the sensitivity [49]. Given that these two competing processes have opposite temperature dependences, their dominance determines the optimum operating temperature of the sensors [50].

Sensitivity can be enhanced by modulating the physical properties of the nanomaterial (e.g., decreasing the substrate inter-electrode distance [51], increasing the porosity [52]), affecting its composition through doping [53], or by using composite materials [54]. While sensor sensitivity can increase by decreasing the size of the grains in the sensing nanostructured materials [55], doing so leads to dense agglomeration and clogging at elevated operating temperatures, thereby decreasing the sensing ability in the long run [56].

Table 3 lists a number of SnO_2_ nanostructured materials, fabricated by different techniques, and the associated sensitivities to 50 ppm CO as determined by the data provided in the respective papers and Eq. 1. Evidently, different fabrication techniques can affect the structure of the nanomaterial building blocks, their crystallinity, and thus their surface functionality [57]. In addition, by decorating the surface of the resulting nanomaterials with well-defined nanoparticles can provide another means of affecting sensor sensitivity as they can change the surface electronic configuration and thus impact the adsorption/desorption of the target gas.

**Table 2:** Threshold limits of common pollutants as set by European Union and US agencies, as well as LoD of best-performing sensors reported in literature

Pollutant	EU Threshold Limits**	US Threshold Limits*** [43]	LoD of best-performing sensor reported in literature	References
CO	10 ppm	9 ppm	1 ppm	[44]
NO_2_	50 ppb	53 ppb	5 ppb	[45]
O_3_	120 ppb	70 ppb	20 ppb	[46]
SO_2_	130 ppb	75 ppb	38 ppb	[47]
CO_2_	N/A	N/A	150 ppb	[48]

**EU air quality standards described in Directive 2008/50/EU

***National Ambient Air Quality Standards (NAAQS) of the US

It should be noted here that while many attempts have been made to improve the sensitivity of MOS gas sensors by affecting the intrinsic (physical and chemical) properties of the sensing materials (i.e., the MOS), external factors including temperature and ambient humidity have shown to be rather dominant in defining overall sensor performance [58]. This warrants for thorough testing and optimization under laboratory and real-life conditions (i.e., at different ambient humidity conditions and oxygen partial pressures) when developing MOS gas sensors for air quality monitoring.

#### Response and recovery times

**Fig. 3:**
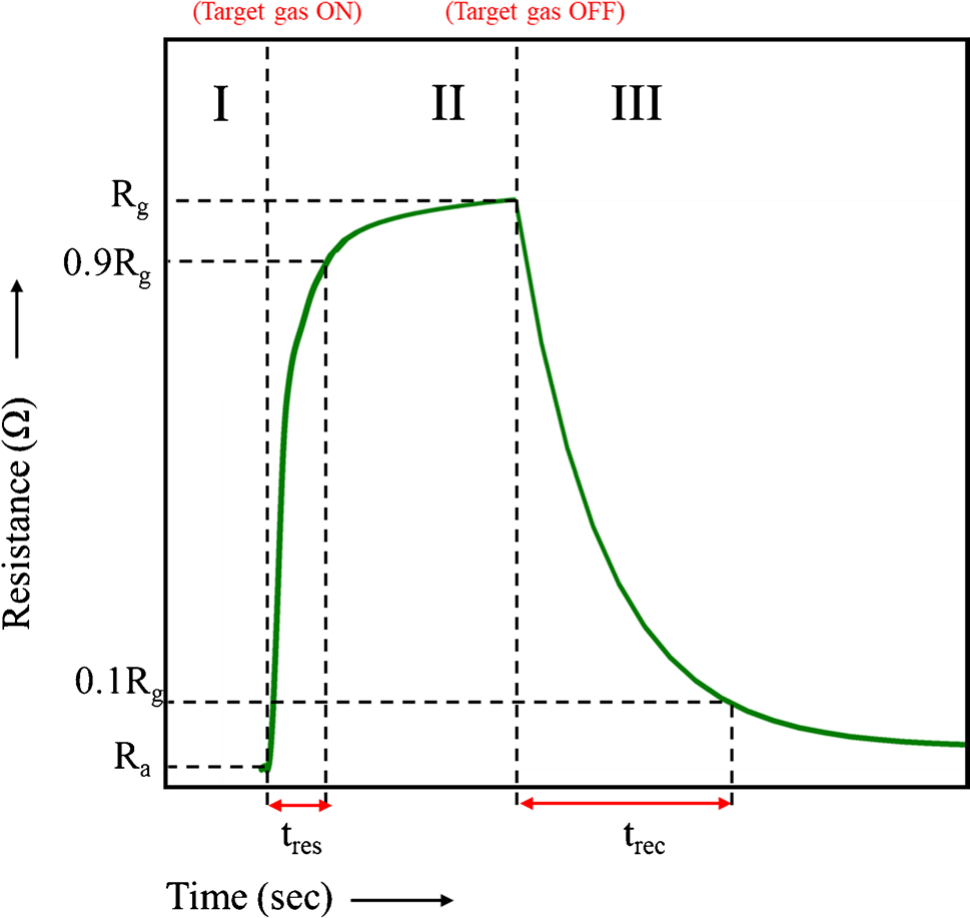
Schematic diagram showing typical response and recovery of the resistance of an *n*-type MOS gas sensor in the presence of an oxidizing target gas. The resistance of the sensing material is stabilized after exposure to the ambient atmosphere and thus is dominated by the adsorption of oxygen species (region I). Upon introduction of the target gas (region II), the resistance begins to rise, reaching a final/saturation value. The time required to reach 90% of the final resistance value is defined as the response time of the sensor (t_res_). Upon removal of the target gas (region III), the resistance of the sensing material decreases, reaching its initial value prior exposure to the target gas. The time required for the resistance of the sensing material to reach values close (typically 10% higher) to the initial value is the recovery time of the sensor (t_rec_)

The dynamic behavior of gas sensors can be expressed by their response and recovery times. The *response time* is the time that the sensor requires to attain a stable signal when exposed to a specific concentration of the test gas. Definitions of the response time vary in the literature. In most cases, it is described as the time required for the resistance οf the sensing material to reach 90% of the saturation value following exposure to the target gas as shown in Fig. 3. The *recovery time* is the time required by the resistance of the sensing material to return to the value it had in the absence of target gas analyte. Typically, the time required to reach a value that is approximately 10% higher than the initial resistance is reported as the recovery time. Reporting the response and recovery times corresponding respectively to the 90 and 10% of the saturation signal of a sensor is practical because most sensors can take several hours to reach the final saturation or base signal due to the relatively slow kinetics of adsorption/desorption.

Similarly to the sensitivity, the response and recovery time of MOS gas sensors can be affected by the intrinsic properties of the materials. Table 4 shows optimal operating temperatures as well as response and recover times of ZnO nanomaterials used for H_2_S sensing. Interestingly, using thin films, but functionalizing them with different metals (Ni, Ti, or Cu) attributing to the materials a different optimal operating temperature as well as improved response and recovery times. Evidently, nanostructuring the thin films can also affect the properties of the material and thus provide another parameter to tune the desired response of the sensors.

**Table 3:** Sensitivities of SnO_2_-based MOS sensors, fabricated by different methods, when exposed to 50 ppm CO. The sensitivities reported are calculated using Eq. 1

Structure and decorating nanoparticles on SnO_2_ nanomaterials	Synthesis method	Sensitivity	References
Nanosheets—Ni/Zn	Hydrothermal process	7.3	[59]
Nanoparticles—Pt	Flame spray	1.5	[60]
Nanowire	Chemical Vapor Deposition	2.5	[61]
Nanopowder—Zn/Fe	Sol gel synthesis	2.0	[62]
SnO_2_ thin films	Pulsed laser deposition	4.0	[63]

#### Selectivity

Selectivity is the ability of gas sensors to identify a target gas among other different gases. For air quality sensors, selectivity is an important parameter because myriad of gaseous species that are present at the ppb or ppt levels in the ambient air could induce changes in the resistance of the MOS in a similar manner with the target gas. Evidently, for species that are typically in high concentrations in the atmospheric environment (i.e., higher than a few tens/hundreds ppm; e.g., CO_2_ and H_2_O) it is much easier to develop selective gas sensors as not many other atmospheric compounds are so abundant [69]. For target species that are in the ppb range, selectivity can be improved by modulating the physical (e.g., metal oxide grain size, operating temperature), or the chemical (e.g., doping, surface functionalization) properties of the MOS, using similar strategies to those for enhancing sensor sensitivity. Adding metallic nanoparticles on the surface of the MOSs can provide another means of improving selectivity, among other properties of the sensors. For example ion reduction can be used to form functionalizing nanoparticles on the surface of metal oxides, similarly to the method reported by Wali et al. [70, 71].

#### Cross-sensitivity

The sensor response to gas analytes other than the target gas is referred to as cross-sensitivity. The term is used complementary to selectivity, as a gas sensor with low selectivity will have high cross-sensitivity, which is typically expressed as a percentage of target gas response. For example, a CO sensor with 25% cross-sensitivity to H_2_ (the interfering gas) will produce a signal that is 25% of the full-scale deflection in the presence of H_2_ without CO. Cross-sensitivities can be positive or negative depending on the MOS surface reaction.

The methods to suppress sensor cross-sensitivity are the same to those for increasing their selectivity. For any sensor designed for air quality monitoring, cross-sensitivities to gases that are rather abundant should be extremely low. Among these gases, H_2_O, which is not a gas pollutant but is rather abundant in the atmospheric environment, can significantly affect the response of the sensors [72, 73]. What is more, the concentration of water vapor in the atmospheric environment can be highly variable depending on location and meteorological conditions, and thus the slightest cross-sensitivity to it may affect the response of the sensors in an unpredictable way. A drier can be installed upstream of the sensor to remove most of the water vapor. However, this needs an efficient design since even variabilities of the order of 1% in relative humidity at 21 ºC can translate to H_2_O vapor concentration fluctuations in the order of a hundred ppm in dry air.

#### Sensor stability

The stability, or reproducibility, of a sensor reflects its ability to produce the same signal over time when exposed to the same concentration of a target gas. It can be distinguished as active or conservative stability [52]. *Active stability* reflects the reproducibility of the sensor characteristics over time, whereas *conservative stability* expresses the retainment of the selectivity and sensitivity of the sensor over time when this is stored under room temperature and ambient humidity [52].

The stability of MOS gas sensors can be affected by a number of factors. Structural changes of MOSs used in gas sensors, which can significantly degrade their overall performance, can be induced by usage over time [74]. Grain growth due to absorption or adsorption of gaseous species can also cause changes in the crystallographic faces [75], the band gap, and the point defects in metal oxides [76].

A property of a sensor that is relevant to stability is drift. This is defined by small changes in the response of the sensor, which are primarily induced by the slow diffusion of adsorbed oxygen species in the lattice of the MOS when this is exposed to identical conditions and the same gas analyte over long periods of time [77]. Long-term drifts can be observed throughout the sensor lifetime, whereas short-term drifts are visible in the first few days of their operation [78].

#### Sensor lifetime

The lifetime of a gas sensor can be expressed in two ways: storage lifetime and operation lifetime. Storage lifetime refers to the time between sensor fabrication and use for the first time. The storage conditions play an important role in the overall lifetime of the sensors. Proper sensor storage should be free of contaminants while temperature and humidity conditions are controlled to the appropriate levels. Operation lifetime refers to the duration between the first time a sensor is used and the time it becomes unfit. Commercial low-cost sensors have typical operation lifetimes of the order of a couple of years.

### Effect of ambient conditions on sensor performance

Designing and building MOS gas sensors that are robust enough and able to measure the concentrations of air pollutants over ranges that are relevant for the atmospheric environment is extremely challenging. Apart from the structural properties of the nanomaterials that can affect sensor sensitivity, selectivity, response time, stability and lifetime, external factors can have a significant impact on their performance. The paragraphs that follow describe how ambient conditions (humidity and temperature) can impact the quality of MOS gas sensors, considering that those are designed to determine air quality.

#### Humidity

Humidity remains one of the main challenges when it comes to optimizing the response of MOS gas sensors, especially when those are designed to be used in the atmospheric environment where the concentration of water vapor can vary substantially over time. Atmospheric water vapor, being the most abundant species in air after nitrogen and oxygen, influences sensing performance because it can easily yield hydroxyl (OH^−^) groups upon adsorption onto the surface of the MOS. This process is in competition with the adsorption of the target gas analyte molecules, thereby affecting sensor performance [79]. The mechanism through which water vapor can affect sensor performance is as follows [35]:

**Table:** 

${\mathrm{H}}_{2}\mathrm{O }\rightleftharpoons {\mathrm{OH}}^{-}+{\mathrm{H}}^{+}$	Reaction 9
${\mathrm{H}}_{2}\mathrm{O }(\mathrm{gas})+2\left(\mathrm{M}+{\mathrm{O}}_{0} \right)\leftrightarrow 2\left( {\mathrm{M}}^{\updelta +}-{\mathrm{OH}}^{\updelta - }\right)+{\mathrm{V}}_{\mathrm{O}}^{2+}+2{\mathrm{e}}^{-}$	Reaction 10
${\mathrm{H}}_{2}\mathrm{O }(\mathrm{gas})+\left(\mathrm{M}+{\mathrm{O}}_{0} \right)\leftrightarrow \left( {\mathrm{M}}^{\updelta +}-{\mathrm{OH}}^{\updelta - }\right)+{(\mathrm{OH})}_{\mathrm{o}}^{+}+{\mathrm{e}}^{-}$	Reaction 11

Here M and ${\mathrm{O}}_{0}$ indicate the lattice metal atom and oxygen, whereases V_O_ and ${(\mathrm{OH})}_{\mathrm{o}}^{+}$ are the oxygen vacancy and the hydroxyl group embedded in the MOS lattice, respectively.

**Fig. 4:**
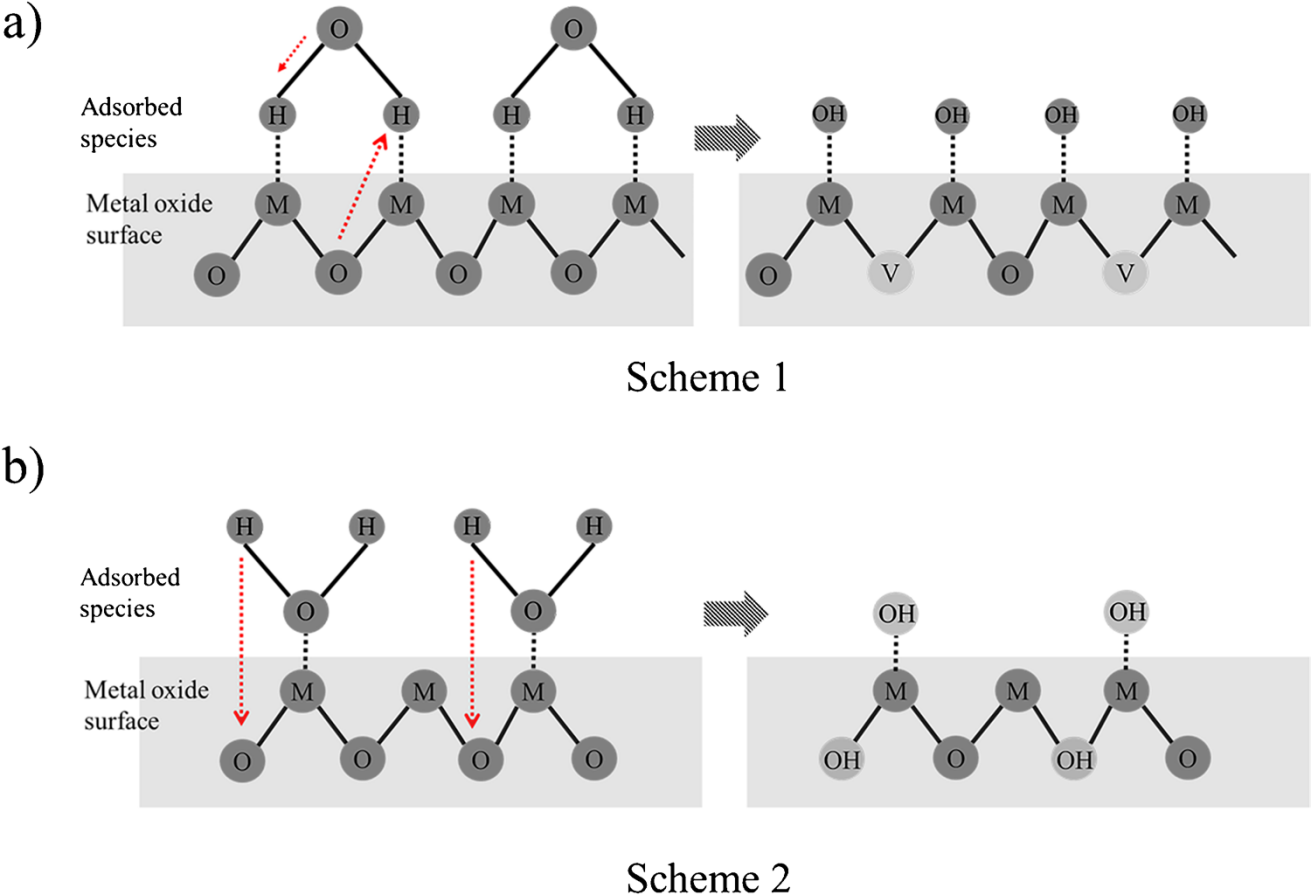
Iillustration showing the interaction of adsorbed water molecules on the surface of a metal oxide, which can deteriorate the performance of gas sensors [81].Water molecules can either adsorb on the metal oxide surface via their (**a**) H or (**b**) O atoms. In the first case, adsorbed H atoms form OH-M bonds on the surface, extracting O atoms from the metal oxide surface and thus create O vacancies (V). In the second case, when water molecules approach the MOS surface via the O atoms, the H of the water molecule chemically interact with the MOS lattice O atoms, incorporating an OH group within the metal oxide surface. Key: M: metal atom; O: oxygen atom; V: oxygen vacancy; OH: Hydroxyl group; H: Hydrogen atom

A schematic illustration of water vapor-MOS interaction is provided in Fig. 4. Water molecules can dissociate on the metal oxide surface by two possible mechanism [80]. In the first case, MOS lattice oxygen atoms from the surface are extracted by the adsorbed hydrogen species, creating oxygen vacancies. At the same time, the surface is decorated with chemisorbed OH groups. In the second case, the oxygen atoms of the water molecule can interact with the MOS surface enabling hydrogen atom incorporation into the MOS surface. In both cases, the surface layer has few metal oxide sites occupied by adsorbed OH^−^ ions, which block the direct interaction with the target gas molecules. What is more, the interaction of hydroxyl ions with adsorbed oxygen species decreases the metal oxide baseline resistance, and consequently the overall sensitivity of the sensor [72].

High humidity aging is one technique used to prevent the degradation of sensors in the presence of water vapor. The sensing layer loaded with an additional noble element (e.g., Pt or Au) is exposed to high concentrations of water vapor (e.g., 90% relative humidity) for a couple of weeks, making the sensor humidity indifferent in a certain range of operating conditions. During this process, the sensing film forms strong bonds with hydroxyl ions, reducing the number of reversible water absorption sites, which in turn makes the sensor resistant to humidity.

Humidity effects on the overall sensor performance can also vary depending on the target gas species for a given MOS. For instance, WO_3_ nano-powders synthesized by liquid-based methods (from tungstic acid) are humidity independent to NO_x_, but are affected by humidity when sensing H_2_S [82]. This unique characteristic can be attributed to the molecular structure of the gas species involved. The structural configuration of H_2_S and H_2_O are very similar (bent geometry), hence they can compete for the same adsorption sites on the MOS surface. On the other hand, nitrogen oxides, which have a linear (for NO) or a trigonal planar (for NO_2_) structure, do not compete with H_2_O molecules, and thus the sensitivity exhibited by the respective MOS sensors is not dependent on humidity levels as reported by Jiménez et al. [82].

#### Temperature

In principle, the performance of MOS gas sensors is not affected by temperature variabilities as the sensing material is typically maintained at elevated temperatures (of the order of a couple of hundred °C) where they exhibit the maximum sensitivity (cf. previous section). In practice, however, small fluctuations in the operation temperature of the sensor can be induced when a sample flow is passed over the sensing MOS (i.e., when the sensor employes active sampling), thereby inducing potential instabilities in the dissipated heat. Dong et al. pointed out the effect of sample gas velocity on the sensor surface temperature and the effect on surface chemical reactions [83]. With higher velocities, the elevated temperature of the MOS surface drops, thereby affecting gas sensitivity. This is expected as surface chemical reactions are temperature-dependent, and thus define the sensitivity of the sensors as explained above (cf. Fig. 2).

MOS gas sensors operated at room temperature have also been proposed and investigated, as they can provide systems that have a low power consumption given that no heating of the sensing material is required [84]. Such sensors, however, are more prone to variabilities of the ambient temperature as typically the operation temperature is not controlled. To overcome this limitation, MOS gas sensors operating at room temperature must be maintained under well-controlled conditions.

## Synthesis and structure of MOS gas sensor nanomaterials

A wide range of methods have been used to synthesize metal oxide nanostructured materials for applications in gas sensing, with each one attributing sensor characteristics that range widely. The properties of the resulting materials depend strongly on their composition and structure, which in turn are determined by the nanoparticle building blocks they consist of. Particle size, morphology and crystal structure are key factors controlling overall gas sensor performance.

Nanomaterial synthesis techniques can be classified either as top-down or as bottom-up approaches. Top down approaches initially start with a macroscopic substance on which nanoscale structures can be created through subsequent removal of material. Common top down approaches include e-beam lithography, photolithography, milling and dry or ion/plasma etching. Top-down processes are typically associated with high manufacturing throughput, but control over surface morphology is limited. Moreover, these approaches typically utilize sophisticated fabrication techniques that are not favorable for cost-effective and/or large-scale industrial production required for a number of applications including gas sensing [85].

In bottom-up approaches, the nanomaterials are built up atom-by-atom and/or block-by-block. Nanomaterials are formed by synthesizing nanoparticle building blocks on surfaces by depositing vapor molecules (in the gas phase) or ions (in the liquid phase). Atoms/ions are stacked together to give rise to crystal planes or atomic clusters, which can further grow to larger particles and material structures. These crystal planes and/or clusters eventually give rise to the nanostructure of the sensing material. Alternatively, one can use nanoparticles synthesized either in the gas (aerosol) or in the liquid (colloid) phase, and then deposit them in a controlled way to form the nanomaterials.

**Fig. 5:**
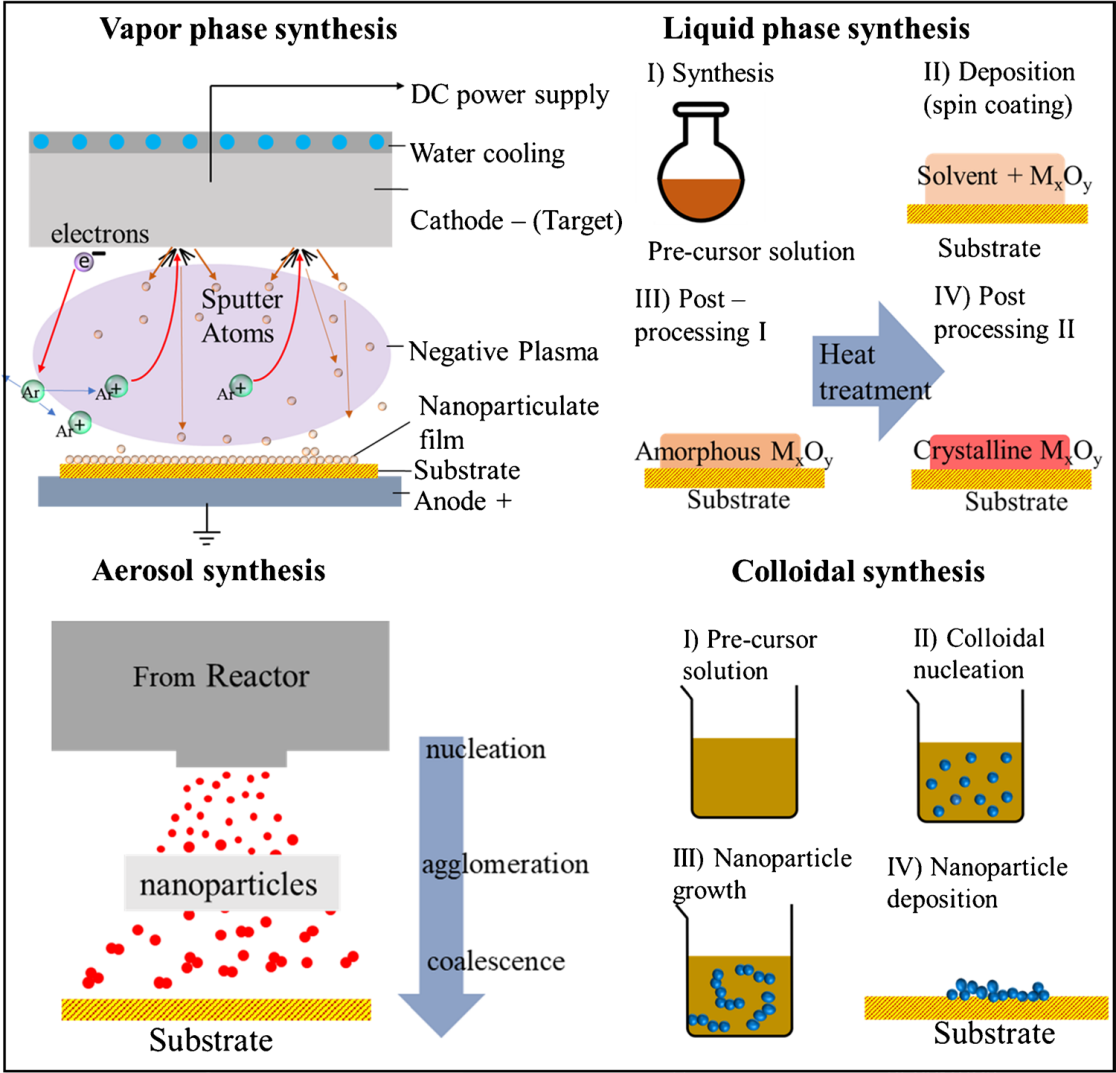
Illustration of the four different bottom-up approaches for nanomaterial synthesis. Vapor phase synthesis methods include formation of vapor atoms or molecules which are subsequently collected on a substrate. Liquid phase methods rely on the formation of atoms/molecules in the liquid phase and on their subsequent deposition on the substrate. Aerosol- or colloidal-based synthesis, employ nanoparticles that are formed in the gas or liquid phase, respectively, before being deposited on a substrate

Bottom up approaches can be broadly classified into *vapor-phase*, *liquid-phase*, *aerosol-based*, and *colloid-based* techniques (cf. Fig. 5). Common bottom-up methods include sol–gel, supercritical fluid synthesis, electrospinning, biologically-assisted synthesis, electrodeposition, spray pyrolysis, flame synthesis, chemical and physical vapor deposition, as well as spark and arc ablation.

### Vapor-phase techniques

Synthesis methods in which vapor molecules deposit on a substrate to form nanostructured building blocks are typically referred to as gas or vapor synthesis techniques. Supersaturated vapors of material are created inside a reactor, which can be stabilized through deposition onto a surface. Common examples include sputtering, thermal/e-beam evaporation, laser ablation, low pressure chemical vapor deposition, and epitaxy. High purity epitaxial films can be prepared by such techniques. All these techniques are rather flexible and versatile, and can produce polycrystalline or amorphous films depending on the material and the reactor conditions. Vapor phase techniques offer the advantage of providing high purity nanomaterials, with ways to control their crystallography and thus their reactivity. However, they have low throughput and typically involve expensive equipment.

### Liquid-phase techniques

Liquid phase nanomaterial synthesis involves techniques that employ chemical reactions in liquid solvents. Examples of such techniques include atomic layer deposition and template assisted electrodeposition. Compared to vapor phase techniques, liquid-phase methods are easily scalable for industrial applications, relatively inexpensive, and also easy to handle [86]. As temperature and heat dissipation can be better controlled in liquids as compared to gases, liquid-phase methods provide very good control of the size and morphology (including cubes, rectangles, and pyramids) of the resulting particles. Despite these unique advantages, however, they are typically implemented as batch process that have high yields, which can be considered as a downside for certain applications.

### Aerosol-based techniques

In aerosol-based processes, nanoparticles are synthesized, treated and transported in the gas phase, typically at atmospheric pressure. Aerosol nanoparticles can be either neutral or charged depending on the synthesis process and can thus be collected on substrates using either mechanical (inertial impaction, diffusional or thermophoretic deposition) or electrostatic methods. By avoiding solvents, aerosol-based methods produce particles of high purity while at the same time they are environmentally friendly as they do not produce any waste streams. Common techniques involve flame synthesis, laser ablation, spark/arc ablation and glowing wires. Aerosol-based methods usually yield small spherical singlet particles or large agglomerates consisting of spherical primary particles. Additionally, they offer the advantage of controlling the structure (or the porosity) of the resulting nanomaterials depending on the technique selected for deposition (e.g., diffusional deposition, which is gentler, or impaction deposition which is more aggressive).

### Colloid-based techniques

Colloidal processes are well established. In contrast to the solution-phase techniques where the nanoparticles/nanostructures are formed and grow on the substrate, nanoparticles in colloid-based techniques are formed as suspensions in the solvent, yielding a colloidal system. Nanoparticles suspended in liquid media can be stabilized using ligands or surfactants, providing complete control over the desired physical properties of the synthesized material. Examples of colloid-based techniques include chemical precipitation, as well as hydrothermal and sol–gel processes. With efficient temperature and pressure control insoluble precipitates can be formed, yielding metal, metal oxide, organic and pharmaceutical nanoparticles. The reaction conditions are monitored during the process to ensure that nucleation and growth of the nanoparticle building blocks is slow and can form colloidal sols.

Table 5 provides a brief overview of all the above-mention techniques, including short descriptions of their advantages and disadvantages.

**Table 4:** Optimal operating temperatures and response/recovery times of ZnO nanomaterials for H_2_S gas sensing

Structure of nanomaterials	Concentration of H_2_S used (ppm)	Operating Temperature (˚C)	*t*_res_ (s)	*t*_rec_ (s)	References
Colloidal ZnO Quantum dots	50	25	16	820	[64]
ZnO comb-like	0.1	25	48	540	[65]
ZnO dendrites	100	25	20	50	[66]
ZnO thin films – Al	600	200	90	209	[67]
MoO_3_/ZnO cages	100	270	13	29	[68]

### Types of MOS sensing materials

MOSs showcase a wide range of materials with electrical behavior that can range from that of insulators (e.g., Al_2_O_3_ and MgO), wide-band/narrow-band gap semiconductors (TiO_2_, SnO_2_, and Ti_2_O_3_, respectively) and metal-like conductors (V_2_O_3_, Na_x_WO_3_, and ReO_3_). They can also be classified as transition and non-transition MOSs [105]. Non-transition MOSs are expected to be relatively inert due to their limited oxidation states, whereas transition MOSs are more sensitive to changes in the ambient conditions. Due to their limited performance at high operation temperatures and relatively poor electroconductivity, however, non-transition MOSs are in general less popular for use as gas sensing materials [106]. That said, they can exhibit enhanced sensing performance for specific target gases as a result of their superior catalytic properties, especially when combined with other metals or metal oxides [107].

In fact, catalytic activity at the surface of MOSs can enhance the sensitivity and selectivity of the end sensors. In this respect, composite metal oxides (e.g., tin dioxide/zinc oxide or tin dioxide/indium oxide) whose behavior towards detecting specific target gases is complementary, offer higher sensitivity and faster kinetics as compared to single metal oxides [108]. Considering also that catalytic reactions are by nature temperature-dependent, producing sensing materials that rely on the catalytic activity on their surfaces can also provide another great means of tuning sensor sensitivity and/or selectivity. We should highlight here that catalytic activity is an important but not a determining factor; in other words, it can improve the performance of the sensors, but not define it.

Other properties of the sensing materials that are important for identifying/selecting the most appropriate for specific target gases include their band gap and type. A high band gap (larger than ca. 3 eV) in combination with low activation energy for the chemical reactions responsible for gas sensing mechanisms is ideal for sensing applications. This is because for higher band gap MOSs the effect of surrounding temperatures on sensor performance is reduced [109]. The type of MOS (*p*- or *n*-type) also plays a role. The most popular MOS materials (i.e., SnO_2_, TiO_2_, WO_3_, ZnO) are *n*-type as they have a number of advantages including that they are thermally more stable and can work at lower partial pressures of ambient oxygen [77]. Common *p*-type oxides (e.g. iron oxides) are typically used to form nanocomposites with *n*-type MOSs in order to improve their sensing performance [110].

### Tuning MOS gas sensor performance

The performance of MOS gas sensors depends on the physical and chemical properties of the sensing nanomaterials/nanostructures, which can be tuned during fabrication. The performance of gas sensors is widely affected by the material morphology. The size of the nanoparticles (0D structures) defines the specific surface area available for sensing. Apart from nanoparticle-based materials, recent efforts have focused on the synthesis of materials consisting of MOS nanofibers (1D structures). An advantage of these materials is the improved sensitivity [111]. 2D and 3D nanoparticle-based networks of porous morphologies (e.g., nanosheets) can enable effective and rapid adsorption of target gas species, giving fast response/recovery times even at concentrations down to the ppb range [112]. Each aspect of the gas sensor performance is related to the growth of material morphologies, which is discussed in detail in the following paragraphs and summarized in Table 6.

**Table 5:** Summary of common techniques used for nanomaterial synthesis

	Synthesis Method	Working principle	Advantages	Disadvantages	Materials synthesized	Refs	
TOP**-**DOWN NANOSYNTHESIS	Lithography	Photolithography—a multistep process yielding nanopatterns on a surface by exposure to light. The first step almost always involves coating the surface with a thin layer of polymer called photoresist	High reliability, low temperature, process simplicity	High amounts of waste produced, high operational cost, masks required, limited morphologies	Silicon-based nanomaterials, conductive inks, MOS nanomaterials	[87]	TOP**-**DOWN NANOSYNTHESIS
		E-beam lithography—a process where an electron beam is used for patterning a surface	No mask template is required, high resolution	Slow and expensive process	Metallic nanomaterials, polymers	[88]	
	Milling	Ion beam milling—a process whereby a beam of ions is focused onto a thin film or bulk material until the required nanostructure is attained	Production of precise nanostructures	Intricate machinery involved, low throughput, time consuming process	Metal and carbon nanoparticle	[89]	
		High energy ball milling – a process where bulk material is placed in a mill with milling balls transferring their kinetic energy to break the material into small nanoparticles	High throughput	Contamination, polydisperse nano-powders, noise pollution	Metallic and MOS nanomaterials	[90]	
	Etching	Plasma etching – a dry etching process during which material surface gets exposed to plasma and bombarded by molecules, ions, electrons, and photons stimulating expulsion of material from the surface, and thus leading to nano-structuring	High resolution, easy to control, no liquid chemical wastes	High cost, poor selectivity, potential radiation damage	Silicon-based, metallic, and composite nanomaterials	[91]	
		Wet etching—a process to remove material and carry it away in the liquid phase using acid or bases that dissolve the material to be etched	Low cost, simple process, high selectivity	Chemical contamination, dependence on crystal orientation, undercutting	Silicon-based nanomaterials,metallic and metal oxide nanoparticles, composite materials	[92]	
BOTTOM-UP NANOSYNTHESIS	Chemical Vapor Deposition (CVD)	Processes during which a precursor compound (vapor) is introduced into a reacting chamber in a controlled way with a carrier gas. Upon encountering the substrate (typically maintained at a high temperature), the precursor undergoes a chemical reaction and deposits as a solid film on the substrate	Applied to a wide variety of base materials, coat precision equipment	High temperatures, stresses induced on the substrate creating mechanical instabilities in the deposited films, Use of potentially toxic precursors, pyrophoric or corrosive	Metal oxides, non-oxides, composite materials	[93]	GAS PHASE SYNTHESIS TECHNIQUES
	Physical Vapor Deposition (PVD)	Sputtering—a vacuum based process in which atoms are ejected from target material and deposited on a nearby surface to form films through momentum transfer	Stable, long-life vaporization source, reactive depositions possible with the right gas species, small volume deposition chamber possible	Low production rates, expensive target materials	Metal oxides, non-oxides, composite materials	[94]	
		E-beam evaporation—electron beam is used to vaporize a target material which is transported and condensed onto a surface	Good strength and durability of deposited films, low contamination	High capital cost	Metallic films, carbon nanomaterials, MOS nanomaterials	[95]	
		Laser/pulsed laser ablation—a focused laser beam is used to evaporate the target material which is then condensed onto a surface	Multilayer growth and chemical reaction on substrates due to high energy particles	Non-uniform film thickness, extremely small particles when not desired	Carbon nanomaterials, MOS nanomaterials, core shell nanoparticles, quantum dots	[96]	
	Electrohydrodynamic spray deposition (Electrospray)	A solution-based method in which a precursor solution is passed through a high-voltage tube forming a spray of charged droplets. The droplets are evaporated on their path to a grounded electrode/substrate where the resulting nanoparticles are deposited	Single-step process, low cost, low amounts of byproducts produced	Low throughput, use of cross-linking agents in pre-cursors	Metal oxides, non-oxides, composite materials	[97]	LIQUID PHASE SYNTHESIS TECHNIQUES
	Electrospinning	A subcategory of electrospray, producing nanofibers rather than nanoparticles	Simple instrumentation, continuous process	Jet instability, toxic precursors	Polymers, ceramic-based materials	[98]	
BOTTOM-UP NANOSYNTHESIS	Green synthesis (biological)	A process whereby organisms such as algae or fungi holding metal ions form nanoparticles through a series of intra and extra cellular activities, making these organisms the bio-factories	Environment friendly, controllable growth, stable nanomaterials	Wide range pf particle sizes, slow process	Metal and carbon-based nanoparticles, polymers	[99]	
	Spark Ablation	A process whereby vaporous produced by repeated sparks on an electrodes are cooled rapidly to form atomic clusters, which further grow by condensation and coagulation to create nanoparticles	Relatively low cost as it avoids the use of expensive energy source. Avoids the contamination of the material through surfactants and solvent impurities	Limited particle production rates, agglomerated particles	Metallic and metal oxides nanoparticles, intermetallic alloyed nanoparticles	[100]	AEROSOL TECHNIQUES
	Arc discharge	A process where an electric arc formed between two electrodes leads to the formation of plasma, producing vapors from the electrode material that subsequently cool down and nucleate to nanoparticles	Simple method that produces consistent high-purity nanomaterials. High throughput production that can be scaled up	Production of highly agglomerated particles	Metallic and semiconducting nanoparticles, alloy nanoparticles, quantum dots	[101]	
	Laser /Flame Spray Pyrolysis	A family of processes during which a solution is atomized inside a reactor where the resulting droplets undergo evaporation, solute concentration, and thermolysis to give porous nanomaterial. Either lasers or flames can be used as the source of thermal energy in such systems	Effective preparation of powders consisting of ultrafine and spherical grains/particles. Good reproducibility in terms of particle size and quality	High cost associated with production of spherical and ultra-pure particles	Metallic and metal oxide nanoparticless, metal composites	[102]	AEROSOL TECHNIQUE
	Hydrothermal route	A process where nanomaterials are fabricated in a closed process system flowing in an aqueous solution above 100 °C. Process parameters that are usually controlled include initial pH of the medium, duration of the process, as well as pressure and temperature conditions	Environment friendly, versatile functionality, controllable particle morphology	High cost of autoclaving, safety issues	Metallic, metal oxide, and ceramics nanoparticles	[103]	COLLOIDAL SYNTHESIS TECHNIQUE
	Sol–gel	A wet chemistry processing method where colloidal particles in liquids undergo gelation, forming interconnected long polymeric chains. Solution is eventually dried to form the required nanomaterial	Homogeneous materials, low operating temperatures, inexpensive instrumentation	High product purity is affected by sol–gel matrix components, expensive metal-based reactants	Metal and metal oxide nanoparticles, metal composites, carbon supported nanoparticles	[104]

**Table 6:** Summary of the synthesis processes that can be employed to produced MOS nanomaterials of different classes. The minimum structure sizes reported for metal oxide gas sensors for different morphologies is listed. The material structures are classified according to the type of the building blocks (0D, 1D, 2D, or 3D), which can attribute specific characteristics to the resulting nanomaterial. Zero dimensional (0D) refers to building blocks with all dimensions in the nano-range, while three dimensional (3D) refers to building blocks with all three dimensions larger than the nano-range (> 100 nm)

Dimensions	Nanostructured features of the MOS material	Synthesis processes	Examples of Structure Sizes
0D	Nanoparticles, nanoclusters, nanocrystals, quantum dots	Zero dimensional nanomaterials have all dimensions within less than 100 nm. They can be synthesized by physical (e.g., thermal evaporation, sputtering or lithography) or chemical (e.g., template method for core–shell nanoparticles, hydrothermal process, aerosol assisted CVD) processes	5 nm nanocrystals [116] 30 nm nanoparticles [24], 80 nm nanocages [117] with wall thickness 5 nm
1D	Nanowires, nanotubes, nanorods, nanoribbons	Sputtering and thermal evaporation techniques have proved reliable for high quality nanostructures with 1 dimension above the nanoscale. Spray pyrolysis has been used to grow ZnO:WO_x_ nanowires. A chemical route involving electrochemical methods can be employed for MnO_2_ nanowire-nanotubes growth. Sol–gel technique can also be used for its simplicity and flexibility. Scaled up CVD is employed at industrial scale for production of nanowires. Laser pyrolysis is employed for giving different sizes/shapes to TiO_2_ nanomaterials	50—150 nm nanorods with 15 µm length[118] 80 × 20 × 30 nm nanoribbons[119]
2D	Nanosheets, nanowalls, nanodisks, nanobelts, thick films, thin films	Two dimensional nanostructures include structures that have two dimensions above the nanoscale. Thermal evaporation has been used for growth of SnO_2_ nanodisks and Ga_2_O_3_ nanosheets. ZnO nanoleafs can be grown through pulsed laser ablation in a liquid. Chemical routes can also be employed to produce array of MnO_2_ nanowalls. Hydrothermal process has been reported for fabricating NiO nanoplates	Nanosheet thickness 1.2 nm, over an area of (300–500 nm) [120]
3D	Spring-like nanocoils, nanoflowers, bridge structures	Fabrication techniques are used to control the alignment of structures during growth and morphology. Sputtering offers precise morphology control. Lithography and arc discharge are other reported methods used to obtain free-form structures. Low cost electrochemical routes offer high purity 3D nanoflowers. ZnO and SnO_2_ nanostructures can be grown through hydrothermal processes	Micron sized Nanoflowers made from < 100 nm nanowires [121],

The LoD of MOS gas sensors can be improved by enhancing the adsorption of the target gas onto the sensing nanomaterials. This can be achieved by increasing the porosity of the sensing materials through controlling the size of the nanoparticle building blocks and also the way those are deposited on the substrate. Sensor selectivity can be improved using two approaches as proposed by Bochenkov et al. [52]. The first approach involves optimizing the operating temperature of the gas sensors that can enable preferential adsorption of target gas molecules on the metal oxide surface. The second approach involves use of gas sensor arrays from which the signals produced from all their elements can be analyzed in order to distinguish among several gas molecules in a mixture.

The sensitivity of MOS gas sensors is strongly dependent on the size of their grains. The relative thickness of the space charge layer to the grain size defines whether the sensing mechanism is controlled by the size of the grains, the size of the grain boundaries, or the thickness of the necks connecting the grains [113]. Grain size and morphology can be controlled during fabrication by adjusting a number of technique-dependent parameters. For example, in wet-chemistry approaches, the size of the resulting grains can be tuned by controlling liquid phase concentrations, synthesis temperature, presence/absence of surface modifiers, and reactor residence time. Similarly, the thickness of the space charge layer is governed by the properties of the material itself. A high concentration of dopants or the magnitude of the voltage applied on the MOSs during sensors operation can affect the width of depletion region.

The stability of MOS gas sensors can also be tuned by controlling the synthesis of the sensing materials. Nanomaterial post-treatment, including isothermal annealing with embedded noble metals (e.g., palladium or platinum) can improve thermal stability and crystallite growth rate of gas sensor material [114]. For example, Gaury et al. [115] grew WO_3_-based nanowires out of a tree-like nanostructured WO_3_ thin film after depositing KOH and annealing, thereby improving connectivity between the tree-like structures and affecting the conductivity of the resulting materials. Such post treatment techniques can apply to nanomaterials produced with any of the MOS synthesis methods described above.

## State-of-knowledge and future perspectives

MOS gas sensors provide attractive candidates for air quality monitoring. Key requirements for air quality gas sensors is to have low LoD (in the order of a ppb for most pollutants), high selectivity, and fast response/recovery times as discussed in the “Performance of the MOS sensors” section.

Nanomaterials for MOS gas sensors can be synthesized by both top-down and bottom-up approaches using physical or chemical processes. Vapor-phase and aerosol-based methods are known to produce high purity nanoparticle building blocks and consequently nanomaterials due to the absence of solvents, but have a lower throughput as compared to solution-phase/colloidal techniques. Vapor phase techniques by virtue of being operated under vacuum provide opportunities for well-controlled contaminant-free nanofilm growth, crystal growth of single-component materials, or materials of uniform stoichiometry in the nanoscale [122]. Aerosol techniques yield unique filamented morphologies and possibilities to create non-equilibrium metastable phases that can be challenging for other methods [123]. At the same time, they enable easy nanomaterial handling and possibilities for scaling up by virtue of being continuous processes.

Colloidal methods, on the other hand, offer better control of particle morphology, and can be used to make cube-, rectangle- and pyramid-shaped nanoparticles [124]. While several vapor techniques are limited to temperature-resistant substrates, as they require elevated local substrate temperature during deposition (e.g. direct current sputtering), colloidal approaches are flexible in producing both organic and inorganic substrates; something that is true also for the aerosol techniques that are also independent of substrate type, as nanoparticle deposition can be separated from the synthesis process, and carried out at room temperature in case the temperatures for the latter are high (e.g., in flame reactors or spark discharge generator).

LoD, sensitivity, selectivity, and response/recovery times for MOS gas sensors, can be tailored by controlling material characteristics (i.e., composition and size of the grains, as well as overall morphology and porosity of the nanomaterials) during the synthesis process; e.g., by adding surfactants or controlling nanoparticle growth [125]. Post-synthesis steps, such as annealing [126] or functionalization [127], can also be employed to tune the MOS material properties (e.g., post-synthesis stabilization of nanoparticles in an organic solvent [128], or thermal treatment to improve material magnetic properties [129]), providing a wide range of options for sensors that can meet demands in air quality monitoring.

One of the key properties that can enable a sensor for use in air quality monitoring is the LoD. The majority of MOS gas sensors have LoD values in the ppm range, whereas most of gaseous pollutants present in the atmospheric environment are in the ppb or ppt levels. This warrants for further efforts aiming at reducing the LoD of current MOS gas sensors, and thus qualify them for air quality monitoring. LoD of MOS sensors can be improved with increasing the porosity of the sensing materials, thereby providing a larger sensing surface area for gas adsorption and detection in the sub-ppm range [130]. Apart from having low LoD values, highly porous materials and thin films having thicknesses down to a few nanometers also exhibit very fast response kinetics [131].

Air quality monitoring sensors are subjected to a wide range of gaseous species, and thus selectivity is another key property that can define the effectiveness in the field. The selectivity of MOSs towards a specific gassous species can in principle be tuned by employing specific catalytic nanoparticles (e.g., Au and Ru) [132]. This requires use of synthesis methods that can allow incorporation of such catalytic nanoparticles in the MOS sensing material. Alternatively, use of distinct sensing material arrays on a single chip (commonly referred to as an electronic nose), where each material yields a variant signal when exposed to the same sample gas, can be used to determine the concentration of more than one gassous pollutants. Electronic noses, which are typically operated in combination with sophisticated algorithms that can disentangle the signals produced by the different sensing materials on the chip in order to estimate the concentration of specific gases, provide a promising approach as it can circumvent issues of selectivity for each sensing material on the array.

Humidity in most cases has a negative effect on metal oxide sensor functioning as already discussed in the “Effect of ambient conditions on sensor performance” section. While some sensors become insensitive at high relative humidity (RH) values [133], others simply exhibit slower response [134]. A possible solution is to use a drier/desiccant, which is common practice in air quality monitoring. Although driers typically employed in such measurements do not make the samples completely dry (i.e., H_2_O-free), they can reduce the RH from ambient down to a few percent. In that range, a 1% change in RH at 21 ˚C translates to changes in H_2_O concentration of the order of a hundred ppm. MOSs that can withstand humidity fluctuation in that range, would therefore showcase higher sensitivity and faster response times.

Long periods of use of MOS gas sensors can diminish their performance (sensitivity, stability, selectivity) leading to signal drifts. Considering the low cost of the MOS gas sensors, which allows for frequent replacements, this may not be seen as the main drawback for their use in air quality monitoring. Nevertheless, ways to suppress sensor drifts over time can be realized (i) by using innovative nanomaterial architectures and metal oxide doping [135], as well as (ii) by employing sophisticated algorithms for adjusting the recorded signals [136].

Overall, MOS gas sensors provide an attractive solution for low-cost measurements of air pollutants. That said, further research and development efforts are needed to improve key sensor properties (e.g., LoD and selectivity) that can enable this technology for air quality monitoring. Such improvements can be made by selecting appropriate synthesis methods that allow tuning the properties of MOS materials in the nanoscale. At the same time, use of arrays of different sensing materials on a single chip, in combination with sophisticated algorithms for converting the signals to concentrations of specific gases, can qualify MOS gas sensor technology for distributed air quality monitoring.
